# Phylogenomic data resolved the deep relationships of Gymnogynoideae (Selaginellaceae)

**DOI:** 10.3389/fpls.2024.1405253

**Published:** 2024-07-16

**Authors:** Jing Zhao, Zhao-Rong He, Shao-Li Fang, Xu-Ke Han, Lu-Yao Jiang, Yu-Ping Hu, Hong Yu, Li-Bing Zhang, Xin-Mao Zhou

**Affiliations:** ^1^ School of Ecology and Environmental Science, Yunnan University, Kunming, Yunnan, China; ^2^ School of Life Sciences, Yunnan University, Kunming, Yunnan, China; ^3^ Missouri Botanical Garden, St. Louis, MO, United States; ^4^ Chengdu Institute of Biology, Chinese Academy of Sciences, Chengdu, Sichuan, China

**Keywords:** conformation, lycophyte, phylogenetic conflict, phylogenetic signal, plastomes

## Abstract

The unresolved phylogenetic framework within the Selaginellaceae subfamily Gymnogynoideae (ca. 130 species) has hindered our comprehension of the diversification and evolution of Selaginellaceae, one of the most important lineages in land plant evolution. Here, based on plastid and nuclear data extracted from genomic sequencing of more than 90% species of all genera except two in Gymnogynoideae, a phylogenomic study focusing on the contentious relationships among the genera in Gymnogynoideae was conducted. Our major results included the following: (1) Only single-copy region (named NR) and only one ribosomal operon was firstly found in *Afroselaginella* among vascular plants, the plastome structure of Gymnogynoideae is diverse among the six genera, and the direct repeats (DR) type is inferred as the ancestral state in the subfamily; (2) The first strong evidence was found to support *Afroselaginella* as a sister to *Megaloselaginella*. Alternative placements of *Ericetorum* and *Gymnogynum* were detected, and their relationships were investigated by analyzing the variation of phylogenetic signals; and (3) The most likely genus-level relationships in Gymnogynoideae might be: ((*Bryodesma*, *Lepidoselaginella*), (((*Megaloselaginella*, *Afroselaginella*), *Ericetorum*), *Gymnogynum*)), which was supported by maximum likelihood phylogeny based on plastid datasets, maximum likelihood, and Bayesian inference based on SCG dataset and concatenated nuclear and plastid datasets and the highest proportion of phylogenetic signals of plastid genes.

## 1 Introduction

Selaginellaceae, the largest family in lycophytes, are nearly cosmopolitan and are estimated to contain 700–800 species (Jermy, 1986, 1990; Tryon and Lugardon, 1991; Zhou and Zhang, 2015; PPG I, 2016; Zhou et al., 2016; Weststrand and Korall, 2016a, b; Zhou and Zhang, 2023) but may contain up to 1000 species. Diverged from their closest living relatives ca. 383 million years ago in the Devonian, they were hyper-diverse already in the Cretaceous based on fossil records and molecular dating (Thomas, 1992, 1997; Kenrick and Crane, 1997; Korall et al., 1999; Taylor et al., 2009; Arrigo et al., 2013; Klaus et al., 2017; Morris et al., 2018; Schmidt et al., 2020, 2022). Selaginellaceae are an ideal example of morphological stasis in plants, and both extant species and fossil materials have morphological stability over hundreds of millions of years (Schmidt et al., 2022). Because of the high intraspecific and low interspecific variability, Selaginellaceae are difficult to identify, and how to subdivide the family has frequently been contentious (e.g., Jermy, 1986, 1990; Zhou and Zhang, 2015; Zhou et al., 2016; Weststrand and Korall, 2016a, b; Zhou and Zhang, 2023).

Most recently, using the largest taxon and character sampling and integrating morphological characters, geographical distribution, Zhou and Zhang (2023) recognized seven subfamilies and 19 genera in Selaginellaceae. Here we adopt their new classification and focus on the phylogeny of the subfamily Gymnogynoideae (= “*S.* subg. *Ericetorum*” *sensu* Zhou and Zhang, 2015). Following the new classification, Gymnogynoideae include six genera: *Afroselaginella* (= *S*. sect. *Myosurus* Li Bing Zhang & X.M.Zhou *sensu* Zhou and Zhang, 2015; = *S*. subg. *Exaltatae* Weststrand & Korall p.p. sensu Weststrand and Korall, 2016b), *Bryodesma* (= *S*. sect. *Homoeophyllae* Spring *sensu* Zhou and Zhang, 2015; = *S*. subg. *Rupestrae* Weststrand & Korall *sensu* Weststrand and Korall, 2016b), *Ericetorum* (= *S*. sect. *Lyallia* (Rothm.) Li Bing Zhang & X.M.Zhou *sensu* Zhou and Zhang, 2015; = *S*. subg. *Ericetorum* Jermy *sensu* Weststrand and Korall, 2016b), *Gymnogynum* (= *S*. sect. *Articulatae* (Spring) Li Bing Zhang & X.M.Zhou *sensu* Zhou and Zhang, 2015; = *S*. subg. *Gymnogynum* (P.Beauv.) Weststrand & Korall *sensu* Weststrand and Korall, 2016b), *Lepidoselaginella* (= *S*. sect. *Lepidophyllae* Li Bing Zhang & X.M.Zhou *sensu* Zhou and Zhang, 2015; = *S*. subg. *Lepidophyllae* (Li Bing Zhang & X.M.Zhou) Weststrand & Korall *sensu* Weststrand and Korall, 2016b), and *Megaloselaginella* (= *S*. sect. *Megalosporarum* Li Bing Zhang & X.M.Zhou *sensu* Zhou and Zhang, 2015; = *S*. subg. *Exaltatae* p.p. *sensu* Weststrand and Korall, 2016b).

**Figure 1:**
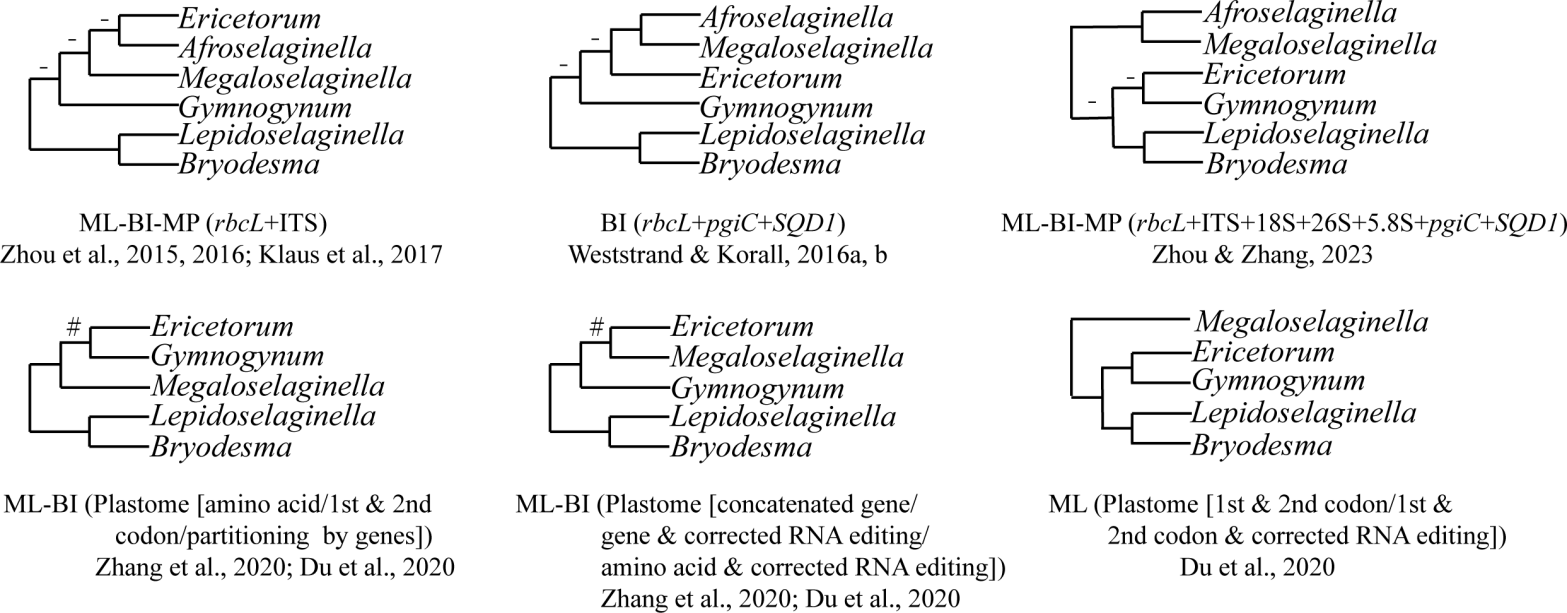
Summary of the topologies among the six genera of Gymnogynoideae among different studies following Zhou and Zhang’s (2023) classification. “-” represents maximum-likelihood bootstrap support values (MLBS) <50%, maximum parsimony jackknife support values (MPJK) <50%, and Bayesian inference posterior probability (BIPP) <0.90; “#” represents maximum-likelihood bootstrap support values (MLBS) <75%.

Gymnogynoideae are well characterized by the often dorsal rhizophores and reticulate megaspores (Zhou and Zhang, 2015; Zhou et al., 2016; Zhou and Zhang, 2023). Species of Gymnogynoideae show high morphological diversity in megaspores, microspores, habit, leaves, strobili, etc (Zhou and Zhang, 2015; Zhou et al., 2016; Weststrand and Korall, 2016a; Zhou and Zhang, 2023). Although the monophyly of the six genera in Gymnogynoideae was each well supported phylogenetically and morphologically (Zhou and Zhang, 2015; Zhou et al., 2016; Weststrand and Korall, 2016a; Zhou and Zhang, 2023), the inter-generic relationships have never been fully resolved (Zhou and Zhang, 2015; Zhou et al., 2016; Weststrand and Korall, 2016a; Zhou and Zhang, 2023). The sister relationship between *Lepidoselaginella* and *Bryodesma* has been confirmed in previous phylogenetic studies using different analyses or datasets (Zhou et al., 2016; Weststrand and Korall, 2016a; Klaus et al., 2017; Du et al., 2020; Zhang et al., 2020a; Zhou et al., 2022; Zhou and Zhang, 2023). However, the relationships among the remaining genera were strongly volatile (**Figure 1**). Zhou et al. (2016) resolved *Gymnogynum* as sister to the remaining genera (except *Lepidoselaginella* and *Bryodesma*) with weak support, and the phylogenetic relationships among *Megaloselaginella*, *Ericetorum*, and *Afroselaginella* were unresolved in maximum likelihood (ML), Bayesian inference (BI), and maximum parsimony (MP) analyses based on the concatenated dataset (*rbcL* and ITS) (**Figure 1**). Weststrand and Korall (2016b) also resolved *Gymnogynum* as the second earliest-diverging lineage in BI analysis, but with low support based on the combined three-region dataset (*rbcL* + *pgiC* + *SQD1*), and resolved *Megaloselaginella* as sister to *Afroselaginella* clade with strong support (**Figure 1**). Recently, the phylogenetic analysis based on plastid *rbcL* and five nuclear markers (18S, 26S, ITS, *SQD1*, and *pgiC*) showed that Gymnogynoideae were comprised by four well-supported caldes: *Ericetorum*, *Gymnogynum*, the *Megaloselaginella + Afroselaginella* clade, and the *Lepidoselaginella + Bryodesma* clade. However, phylogenetic relationships among them were entirely unresolved in maximum likelihood (ML), Bayesian inference (BI), and maximum parsimony (MP) analyses (MPJK and MLBS <50% and BIPP <90%) (**Figure 1**; Zhou and Zhang, 2023).

In recent years, with the boosting of next-generation sequencing, complete or nearly complete plastomes have been largely used to infer the phylogeny of Selaginellaceae (Du et al., 2020; Zhang et al., 2020a; Zhou et al., 2022). Gymnogynoideae included about 130 species with worldwide distribution (Zhou and Zhang, 2015; Weststrand and Korall, 2016b; Zhou and Zhang, 2023). In previous phylogenomic studies, only fewer than eight species in Gymnogynoideae were investigated (Du et al., 2020; Zhang et al., 2020a; Zhou et al., 2022). It is well known that appropriate and extensive taxon sampling is one of the most important determinants to improve the accuracy of phylogeny inferences (Heath et al., 2008). *Afroselaginella* contains about five species (Zhou and Zhang, 2015; Weststrand and Korall, 2016a, b; Zhou and Zhang, 2023), but this genus was never included in previous phylogenomic studies (Du et al., 2020; Zhang et al., 2020a). The inter-generic relationships in Gymnogynoideae were still unsolved or not well supported even by studies based on plastome data. Analyses of the phylogeny of Selaginellaceae with different derived datasets (e.g., amino acid dataset, the first and second codon sites, concatenated unpartitioned dataset, concatenated datasets partitioned by genes or codon positions, and dataset corrected by RNA-edited sites) and analytical methods (ML/BI) were conducted (**Figure 1**; e.g., Du et al., 2020; Zhang et al., 2020a; Zhou et al., 2022; Zhou and Zhang, 2023). However, except *Lepidoselaginella* being constantly resolved as sister to *Bryodesma*, the remaining genera usually showed alternative relationships largely caused by the uncertain placement of *Megaloselaginella*. *Megaloselaginella* was resolved as either sister to the *Gymnogynum* + *Ericetorum* clade (Du et al., 2020: CDS, AA) or sister to the *Lepidoselaginella + Bryodesma + Gymnogynum* + *Ericetorum* clade (Du et al., 2020: codons 1 and 2) or sister to *Ericetorum* (Du et al., 2020: CDSr, AAr). Using the same data processing of codons 1 and 2, Zhang et al. (2020) resolved *Megaloselaginella* as sister to the *Gymnogynum* + *Ericetorum* clade, which was different from the results of Du et al. (2020). In those analyses, the inter-generic relationships received weak to strong support (ML bootstrap support—MLBS = 50% – 100%/Bayesian inference posterior probability—BIPP = 0.73–1) (Du et al., 2020; Zhang et al., 2020a). Different inference methods, samplings, and strategies of data processing strongly affected the resolutions in Gymnogynoideae (**Figure 1**).

In this study, based on the largest taxon sampling so far, we used plastome data and 140 single-copy nuclear genes to further explore the evolutionary relationships in Gymnogynoideae. Multi-datasets were applied to gain insights into the deeper relationships in this subfamily. Our results provide the important insight into plastome-based and nuclear-based phylogenetic relationships in the subfamily and outline the significance for future phylogenomic studies among genera or subfamilies employing the plastomes in Selaginellaceae.

## 2 Materials and methods

### 2.1 Taxon sampling and sequencing

Nine samples were added and sequenced here, plus 24 accessions available in GenBank and our previous work (Zhou et al., 2022) (**Supplementary Table S1**). The total genomic DNA of nine samples was extracted from herbarium specimens. Single-stranded circular DNA libraries were constructed and sequenced on Illumina HISeq 2500 platform using 150-bp pair-end sequencing by Beijing Novogene Technology Co., Ltd. (Tianjin, China). In total, there were 33 samples representing ca. 25 taxa of all six genera in Gymnogynoideae *sensu* Zhou and Zhang (2023), and all six sections of *Selaginella* subg. *Gymnogynum* sensu Zhou and Zhang (2015) correspond to all subgenera in Weststrand and Korall’s (2016b) classification. These samples represent over 90% species of all genera in the subfamily, except *Bryodesma* and *Gymnogynum*. Four other Selaginellaceae outside of Gymnogynoideae were included as part of the ingroup based on the previous works of Zhou et al. (2022) and Zhao et al. (unpubl. data) (**Supplementary Table S1**). Two species of *Isoëtes* were selected as outgroups (Wood et al., 2020) (**Supplementary Table S1**).

### 2.2 Plastome assembling and annotation

Adapter, containing Ns, and low-quality bases from the raw data were first filtered by Fastp v0.12.4 (Chen et al., 2018). *De novo* assemblies were constructed using GetOrganelle v1.7.5 (Jin et al., 2020). Bandage v0.8.1 (Wick et al., 2015) was also used to visualize *de novo* assembly graphs. Complete plastome annotation was performed through the online program Geseq (Tillich et al., 2017). The initial annotation was subsequently inspected and adjusted manually by comparing it with the complete plastome, which was published in our previous study, confirming the start and stop codons and the exon–intron boundaries of genes in Geneious Prime 2019.2.1. A Blastn search was conducted with default parameter settings for any uncertain genes. In addition, all tRNA genes were validated using the online tRNAscan-SE v2.0 service (Chan et al., 2021).

### 2.3 Alignment and phylogenetic inference

The complete plastomes of Gymnogynoideae were acquired in our study. However, previous studies have shown that the plastome of Selaginellaceae has different degrees of gene loss and inversion (Tsuji et al., 2007; Smith, 2009; Xu et al., 2018; Mower et al., 2019; Zhang et al., 2019a, b; Kang et al., 2020; Zhang et al., 2020a; Xiang et al., 2022; Zhou et al., 2022), and it could not be aligned with confidence. In addition, to alleviate the impact of missing data, the Python scripts “get_annotated_regions_from_gb.py” (https://github.com/Kinggerm/PersonalUtilities/) were used to automatically extract 51 protein-coding gene loci and 23 intergenic loci which was shared by all samples. All loci were aligned individually in Mafft v7.450 (Katoh and Standley, 2013) with setting “E-INS-i” and trimmed poorly aligned regions using trimAI v1.3 (Capella-Gutierrez et al., 2009) with setting “automated1”.

We generated four basic datasets, namely: (1) 51 concatenated gene (gene), (2) 23 concatenated intergenic regions (intergene), (3) 74 concatenated gene and intergenic regions (gene_intergene), and (4) first and second codon sites of 51 concatenated gene (codon12). ModelFinder (Kalyaanamoorthy et al., 2017) was used to select the best-fitting likelihood model (**Supplementary Table S2**) for maximum likelihood (ML) and Bayesian inference (BI) under the Corrected Akaike Information Criterion (AICc). ML tree searchers were conducted using IQ-tree v2.1.3 (Nguyen et al., 2015) with 5,000 ultrafast bootstraps (Hoang et al., 2018) analyses in a single run. BI was conducted using MrBayes 3.2.2 (Ronquist et al., 2012). Four Markov chain Monte Carlo chains were run, each beginning with a random tree and sampling one tree every 100 generations of 2,000,000 generations, and the first 25% of samples were discarded as burn-in. Each tree was visualized with their maximum-likelihood bootstrap support values (MLBS) and Bayesian inference posterior probability (BIPP) in Figtree v1.4.3 (Rambaut, 2017).

### 2.4 Quantification of phylogenetic signal for alternative tree topologies

To assess the variation of the phylogenetic signal of each alternative topology of the conflicting nodes, both delta site-wise log-likelihood scores (ΔSLS) and delta gene-wise log-likelihood support (ΔGLS) were used to quantify the distribution of phylogenetic signal for three alternative topologies of *Ericetorum* and four alternative topologies of *Gymnogynum*. For each of the above-mentioned four basic datasets, the analytical methods of Shen et al. (2017) were followed. The average ΔSLS for each locus was separately calculated to avoid the influence of length in the four basic datasets, and standard deviation was used to identify outliers. Boxplot method was used to identify these outlier loci, in which the average ΔSLS values were more significant than the upper bound or smaller than the lower bound of the boxplot, and plotted in OriginPro v2022b (OriginLab Corporation, Northampton, MA, USA). Then, those involved outlier genes were pruned from the above-mentioned four basic datasets in an effort to reduce the conflict at the position of *Gymnogynum* and *Ericetorum* (**Supplementary Table S3**). For the placement of *Gymnogynum*, we generated another four reduced datasets (codon12_RG, gene_RG, intergene_RG, and gene_intergene_RG) after removing the outlier loci of *Gymnogynum* from the basic datasets. We also generated another four reduced datasets of codon12_RE, gene_RE, intergene_RE, and gene_intergene_RE after removing the outlier loci of *Ericetorum* from basic datasets. Finally, another four datasets (codon12_REG, gene_REG, intergene_REG, and gene_intergene_REG) were generated for *Gymnogynum* and *Ericetorum* after removing the outlier genes. Phylogenetic trees were reconstructed using IQ-tree and MrBayes as described previously. The phylogenetic signal was also recalculated, and plots were made using OriginPro.

### 2.5 Assembly of single-copy orthologues and phylogeny reconstruction

HybPiper v1.3.1 (Johnson et al., 2016) was used to assemble single-copy genes (SCGs) from sequenced quality-filtered reads. The sequences of our previous study (Zhao et al., unpublished data) had identified 140 SCGs that were used as target input file for HybPiper. Each gene was aligned individually in Mafft with setting “E-INS-i”, and poorly aligned regions were trimmed using trimAI with setting “automated1”. ModelFinder was used to select the best-fitting likelihood model (**Supplementary Table S2**) for ML and BI under the Corrected Akaike Information Criterion (AICc) based on the concatenated single-copy genes matrix (SCGs). ML tree searchers were conducted using IQ-tree with 5,000 ultrafast bootstrap analyses in a single run. BI was conducted using MrBayes. Four Markov chain Monte Carlo chains were run, each beginning with a random tree and sampling one tree in every 100 generations of 2,000,000 generations, and the first 25% of the samples were discarded as burn-in. In addition, the four basic datasets of plastome were concatenated with single-copy genes matrix (SCGs), respectively. For the four derived datasets (SCGs_codon12, SCGs_gene, SCGs_intergene, and SCGs_gene_intergene) that combined, nuclear and plastid loci datasets were used in the same tree searches (ML and BI) as described previously.

### 2.6 Concordance, character evolution, and phylogenetic networks

The program PhyParts (Smith et al., 2015) was used to identify both plastid loci tree and nuclear loci tree concordance and discordance for each node in the concatenated trees. Each locus tree was reconstructed using ML as implemented by IQ-tree, with the substitution model selected using ModelFinder (set -AICc) and nodal support assessed using 1,000 ultra-fast bootstrap replicates. The results were visualized using the Python script “phypartspiecharts.py” (https://github.com/mossmatters/MJPythonNotebooks). Two discrete characters were reconstructed in their marginal ancestral states in R/phytools (Revell, 2011) with our tip coding states (**Supplementary Table S4**) (model = “ER”). Habit (hydrophytes, xerophytes, mesophytes) and plastome master structures [DR (the repeats region of plastome was direct repeats), IR (the repeats region of plastome was two inverted repeats), NR (the plastome without repeat regions), and DR–IR coexisting (direct repeats and inverted repeats co-existing in plastome)] were scored. They were selected and coded following previous studies (Tryon, 1955; Chu, 2006; Zhang et al., 2013; Zhou and Zhang, 2015; Zhou et al., 2016; Weststrand and Korall, 2016a, b; Zhang et al., 2019a; Zhou and Zhang, 2023, b; Zhang et al., 2020a; Zhou et al., 2022) and our investigations for Selaginellaceae. To explore specific reticulate evolution events and gene flow, a maximum of five reticulation events was set with the command “inferNetworks_MPL” in PhyloNet (Yu and Nakhleh, 2015). An individual SCG tree used as input was generated by IQ-tree with 1,000 rapid bootstrap replicates and then visualized in Dendroscope v3.8.1 (Huson and Scornavacca, 2012).

## 3 Results

### 3.1 Characteristics of plastomes in Gymnogynoideae

All sequenced plastomes of nine samples representing four genera (*Lepidoselaginella*, *Ericetorum*, *Megaloselaginella*, and *Afroselaginella*) in Gymnogynoideae were completely assembled. The plastomes of *L. lepidophylla* and *B. peruvianum* had a typical quadripartite structure composed of a large single-copy (LSC), a small single-copy (SSC), and two inverted repeats (IR), but the remaining species had diverse and unique plastome structures which were divided into four types: DR, IR, NR, and DR–IR. *L*. *lepidophylla* and *B. peruvianum* were IR type with two isomeric forms via a homologous recombination between the two IR copies. The plastomes of all species of *Ericetorum* sequenced were DR type with only one conformation and had a pair of direct repeats (DR) without other short or medium repeats in SC. In *Gymnogynum*, except that the clade A comprised *G. arthriticum* and *G. lingulatum* were DR–IR type with three conformations for both IR and DR in single-copy region (SC), the other clades were DR type with only one conformation. All samples of *Afroselaginella* were NR type and with only one conformation, which was first found in this study. Same as the clade A in *Gymnogynum*, *Megaloselaginella* also has three conformations for both IR and DR in SC. In *Bryodesma*, except that the plastome of *B. peruvianum* was IR type with two conformations for a pair of inverted repeats in SC, the remaining species were DR type.

The plastome features of Gymnogynoideae were strongly different among the six genera (**Supplementary Table S5**). The sizes of the 33 plastomes ranged from 100,119 bp in *Afroselaginella myosurus* (voucher: *C.J. Rothfels et al. 08–183*) to 131,938 bp in *Gymnogynum remotifolium* (voucher: *X.-M. Zhou 722*). The plastome GC content ranged from 50.70% in *Ericetorum pectinatum* (voucher: *F. Rakotondrainibe 6494*) to 56.70% in *G. arthriticum*. *Afroselaginella* has NR type of plastome with only SC. The length of LSC was from 45,276 bp in *E. lyallii* (Zhang et al., 2019a) to 91,273 bp in *Bryodesma peruvianum*. The length of SSC was from 16,732 bp in *G. lingulatum* to 46,373 bp in *G. remotifolium* (voucher: *X.-M. Zhou 722*). The shortest repeat (RC) was 364 bp in *B. peruvianum* (lost in *Afroselaginella*), and the longest was 18,194 bp in *B. rupestre*. For the gene contents, *Afroselaginella* has minimal genes (72), tRNAs (10), and rRNAs (4). Other genera have genes ranging from 80 to 98, tRNAs from 10 to 17, and rRNAs from four to eight, respectively.

### 3.2 Phylogenetic relationships of Gymnogynoideae based on plastid loci

**Figure 2:**
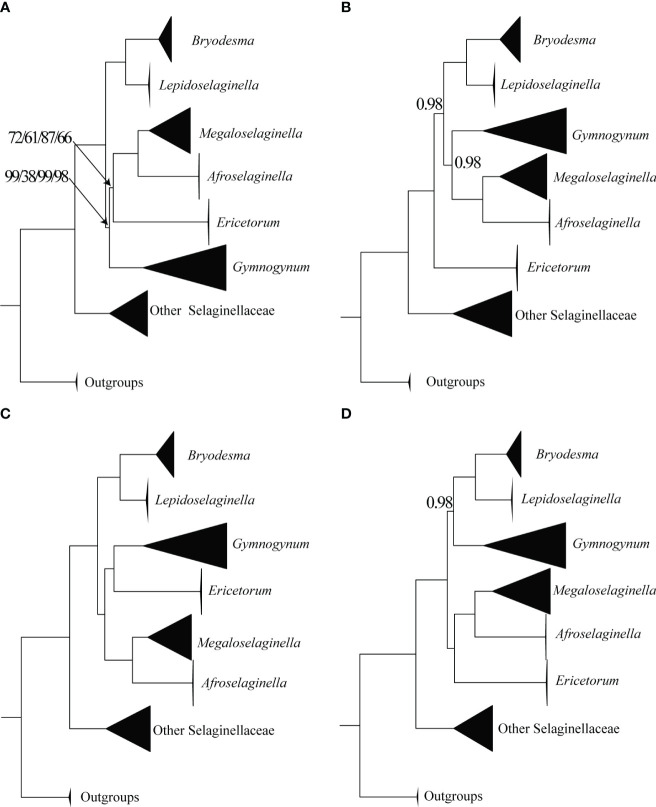
Simplified phylogeny of Gymnogynoideae based on different datasets and methods. **(A)** Maximum likelihood phylogeny based on gene/intergene/gene_intergene/codon12/dataset. **(B)** Bayesian inference phylogeny based on codon12 dataset. **(C)** Bayesian inference phylogeny based on gene and gene_intergene dataset. **(D)** Bayesian inference phylogeny based on intergene dataset. The size of the black triangles was in proportion to the sampled size of individual clades. Maximum-likelihood bootstrap support values (MLBS) and Bayesian inference posterior probability (BIPP) are shown above the branches and are 100/1.0 unless otherwise indicated.

All six genera in Gymnogynoideae were recovered as monophyletic and fully supported in four basic datasets (codon12, gene, intergene, and gene_intergene) in both ML and BI analyses (**Figure 2**; **Supplementary Figures S1**-**S4**). However, four alternative phylogenetic relationships among these six genera were recovered. In all ML analyses based on four basic datasets, *Ericetorum* was sister to the *Megaloselaginella* + *Afroselaginella* clade with medium support (MLBS = 61–87), and *Gymnogynum* was recovered as sister to a clade composed of *Ericetorum* + *Megaloselaginella* + *Afroselaginella* with weak to strong support based on four basic datasets (gene: MLBS = 99, intergene: MLBS = 38, gene_intergene: MLBS = 99, and codon12 dataset: MLBS = 98; **Figure 2A**). In BI analysis, the relationship of the codon12 dataset revealed that *Ericetorum* was sister to other genera of Gymnogynoideae with maximum support (BIPP = 1; **Figure 2B**), and *Gymnogynum* was resolved as sister to a clade containing *Megaloselaginella* + *Afroselaginella* with strong support (BIPP = 0.98; **Figure 2B**). Based on both the gene and gene_intergene datasets, BI analysis revealed that the *Ericetorum* + *Gymnogynum* clade (BIPP = 1.0; **Figure 2C**) was consistently recovered as sister to a clade containing *Megaloselaginella* + *Afroselaginella* (BIPP = 1.0; **Figure 2C**). Comparing the resolutions based on the intergene dataset with BI tree, the sister relationship between *Gymnogynum* and a clade comprised by *Bryodesma* + *Lepidoselaginella* was recovered with strong support (BIPP = 0.98; **Figure 2D**), and *Ericetorum* was sister to a clade composed of *Megaloselaginella* + *Afroselaginella* with maximum support (BIPP = 1.0; **Figure 2D**). Overall, *Ericetorum* had three alternative placements in all ML and BI analyses based on four basic datasets of plastid: T1 ((*Ericetorum*, (*Megaloselaginella*, *Afroselaginella*)), *Gymnogynum*); T2 (*Ericetorum*, other genera of Subfamily Gymnogynoideae); and T3 ((*Ericetorum*, *Gymnogynum*), (*Megaloselaginella*, *Afroselaginella*)) (**Supplementary Figure S5**); *Gymnogynum* had four alternative placements in all ML and BI analyses based on four basic datasets of plastid: T1 (*Gymnogynum*, (*Ericetorum*, (*Megaloselaginella*, *Afroselaginella*))); T2 ((*Gymnogynum*, *Ericetorum*), (*Megaloselaginella*, *Afroselaginella*)); T3 ((*Gymnogynum*, (*Bryodesma*, *Lepidoselaginella*)), other genera of subfamily Gymnogynoideae); and T4 ((*Gymnogynum*, (*Megaloselaginella*, *Afroselaginella*)), *Ericetorum*) (**Supplementary Figure S6**).

**Figure 3:**
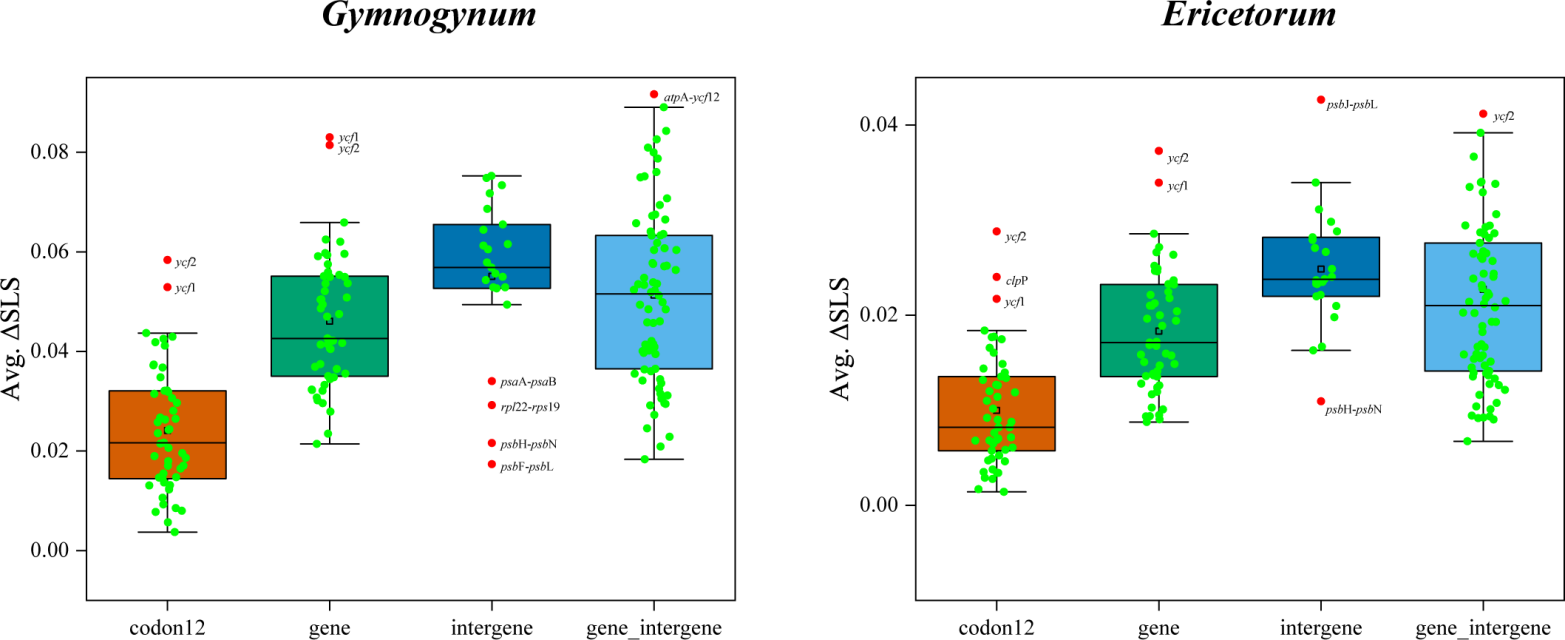
Boxplot was used to define outlier loci and to prune the four basic datasets (codon12, gene, intergene, and gene_intergene). Involved outlier gene of *Gymnogynum* and *Ericetorum* from each dataset (marked with red dot and names).

After the outlier loci from basic datasets of plastid were removed, both ML and BI analyses were reconducted for the alternative topologies of *Gymnogynum* and *Ericetorum*. For the alternative topologies of *Gymnogynum*, the genus involved both codon12_RG and gene_RG removing two outlier loci (*ycf*1 and *ycf*2) from codon12 and gene dataset, intergene_RG dataset removing four outlier loci (*psa*A*-psa*B, *rpl*22-*rps*19, *psb*H-*psb*N, and *psb*F-*psb*L) from gene_intergene dataset, and gene_intergene_RG dataset removing one outlier loci (*atp*A-*ycf*12) from gene_intergene dataset (**Figure 3**; **Supplementary Table S3**). For the alternative topologies of *Ericetorum*, the genus involved codon12_RE dataset removing three outlier loci (*clp*P, *ycf*1, and *ycf*2) from the codon12 dataset, the gene_RE dataset removing two outlier loci (*ycf*1 and *ycf*2) from the gene dataset, the intergene_RE dataset removing two outlier loci (*psb*H-*psb*N and *psb*J-*psb*L) from intergene dataset, the gene_intergene_RE dataset removing one outlier loci (*ycf*2) from gene_intergene dataset to generate (**Figure 3**; **Supplementary Table S3**). All analyses showed that the topologies of ML and BI were each identical to those based on the same datasets with the outlier loci included, but their support values were variable among different datasets (**Supplementary Figures S1**-**S4**).

### 3.3 Conflicting phylogenetic signals of plastid loci

**Figure 4:**
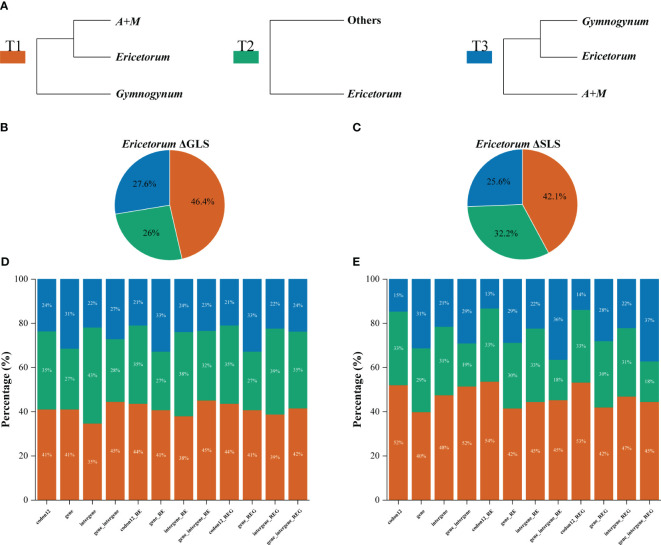
Distribution of the phylogenetic signal for three alternative topological hypotheses at the placement of *Ericetorum* of Selaginellaceae subf. Gymnogynoideae. **(A)** Three alternative topological hypotheses. **(B, C)** The pies summarized the ΔGLS proportion and ΔSLS proportion supported by each alternative topology in the 12 datasets, respectively. **(D, E)** Proportions of ΔGLS and ΔSLS supporting the alternative topologies of three conflicting topologies in each of the 12 datasets. *M*, *Megaloselaginella*; *A*, *Afroselaginella*.

**Figure 5:**
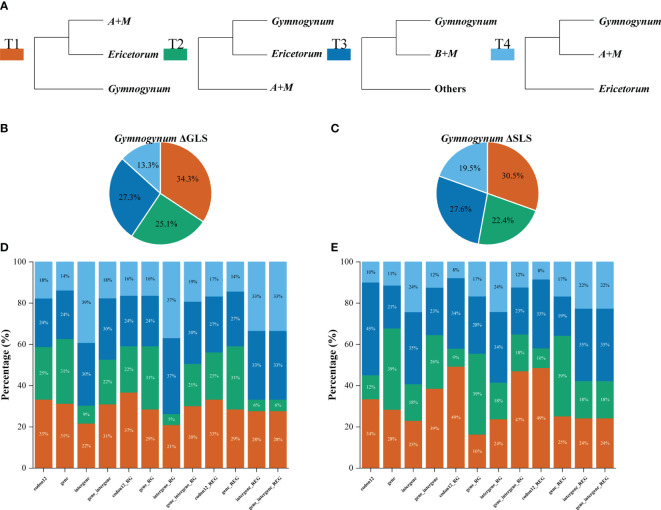
Distribution of phylogenetic signal for four alternative topological hypotheses at the placement of *Gymnogynum* of Selaginellaceae subf. Gymnogynoideae. **(A)** Four alternative topological hypotheses. **(B, C)** The pies summarized the ΔGLS proportion and ΔSLS proportion supported by each alternative topology in the 12 datasets, respectively. **(D, E)** Proportions of ΔGLS and ΔSLS supporting the alternative topologies of three conflicting topologies in each of the 12 datasets. *M*, *Megaloselaginella*; *A*, *Afroselaginella*; *B*, *Bryodesma*; *L*, *Lepidoselaginella*.

The phylogenetic signals for *Gymnogynum* and *Ericetorum* with conflicting topologies as noted above were examined. Both *Gymnogynum* and *Ericetorum* were plotted in the tree, marking the nodes with the alternative positions (**Figures 4A**, **5A**; **Supplementary Figures S5**, **S6**). For *Ericetorum*, intergene dataset supported T2 with the highest proportion of ΔGLS (43%), and 41%–45% loci supported T1 with the highest proportion of ΔGLS based on the remaining three basic datasets (codon12, gene, and gene_intergene dataset) (**Figure 4D**). After the outlier loci were removed, T1 (38%) and T2 (39%) had a similar proportion of ΔGLS support based on intergene_RE and gene_intergene_REG datasets. T1 was still supported with the strongest ΔGLS (41%–45%) with all derived datasets (**Figure 4D**). The examination of ΔSLS values showed the same trend proportions of sites supporting T1 to T3. Both fundamental and derived datasets showed the highest proportion of ΔSLS supporting T1 (42%–54%; **Figure 4E**). In summary, for the 12 datasets of *Ericetorum*, T1 showed a higher support with strongest ΔGLS (46.4%) and strongest ΔSLS (42.1%; **Figures 4B, C**).

Within *Gymnogynum*, the examination of ΔGLS and ΔSLS values showed a greater variation of proportions of loci and sites supporting from T1 to T4 based on different datasets. The codon12 dataset showed the highest proportion of ΔGLS supporting T1 (33%) and supported T1 (37% and 33%, respectively) based on codon12_RG and codon12_REG datasets after the outlier loci were removed (**Figure 5D**). The gene dataset showed a similarly high proportion of ΔGLS supporting T1 (31%) and T2 (31%), but with the highest proportion of ΔGLS supporting T2 (31%) with both gene_RG and gene_REG datasets (**Figure 5D**). However, the intergene dataset supported T4 with the highest proportion ΔGLS (**Figure 5D**) and with a similarly high proportion of ΔGLS supporting T3 and T4 (37% and 33%, respectively) with intergene_RG and intergene_REG datasets (**Figure 5D**). Wtih gene_intergene dataset, T1 had the highest proportion of ΔGLS (31%). T1 and T3 had a similarly high proportion (30%) of ΔGLS with gene_intergene_RG dataset (**Figure 5D**). T3 and T4 had an equally high proportion (33%) of ΔGLS with gene_intergene_REG dataset (**Figure 5D**). Within the qualification of ΔSLS (**Figure 5E**), T2 had the highest proportion of ΔSLS with gene (39%), gene_RG (39%), and gene_REG (39%) datasets (**Figure 5E**). T3 had the highest proportion of ΔSLS with intergene (35%), intergene_RG (34%), and intergene_REG (35%) datasets (**Figure 5E**). When the outlier sites were included, T3 (45%) had the highest proportion of ΔSLS with the codon12 dataset (**Figure 5E**), but both codon12_RG and codon12_REG datasets showed the highest proportion of ΔSLS supporting T1 (49%; **Figure 5E**). T1 had the highest proportion of ΔSLS supporting T1 with gene_intergene and gene_intergene_RG datasets, and T3 (35%) had the highest proportion of ΔSLS with gene_intergene_REG, which was generated by the further removal of outlier loci in *Ericetorum* (**Figure 5E**). In summary, for the 12 datasets of *Gymnogynum*, T1 showed higher support with the strongest ΔGLS (34.3%) and the strongest ΔSLS (30.5%; **Figures 5B, C**).

### 3.4 Phylogenetic inference based on single-copy nuclear genes and combined plastid and nuclear datasets

**Figure 6:**
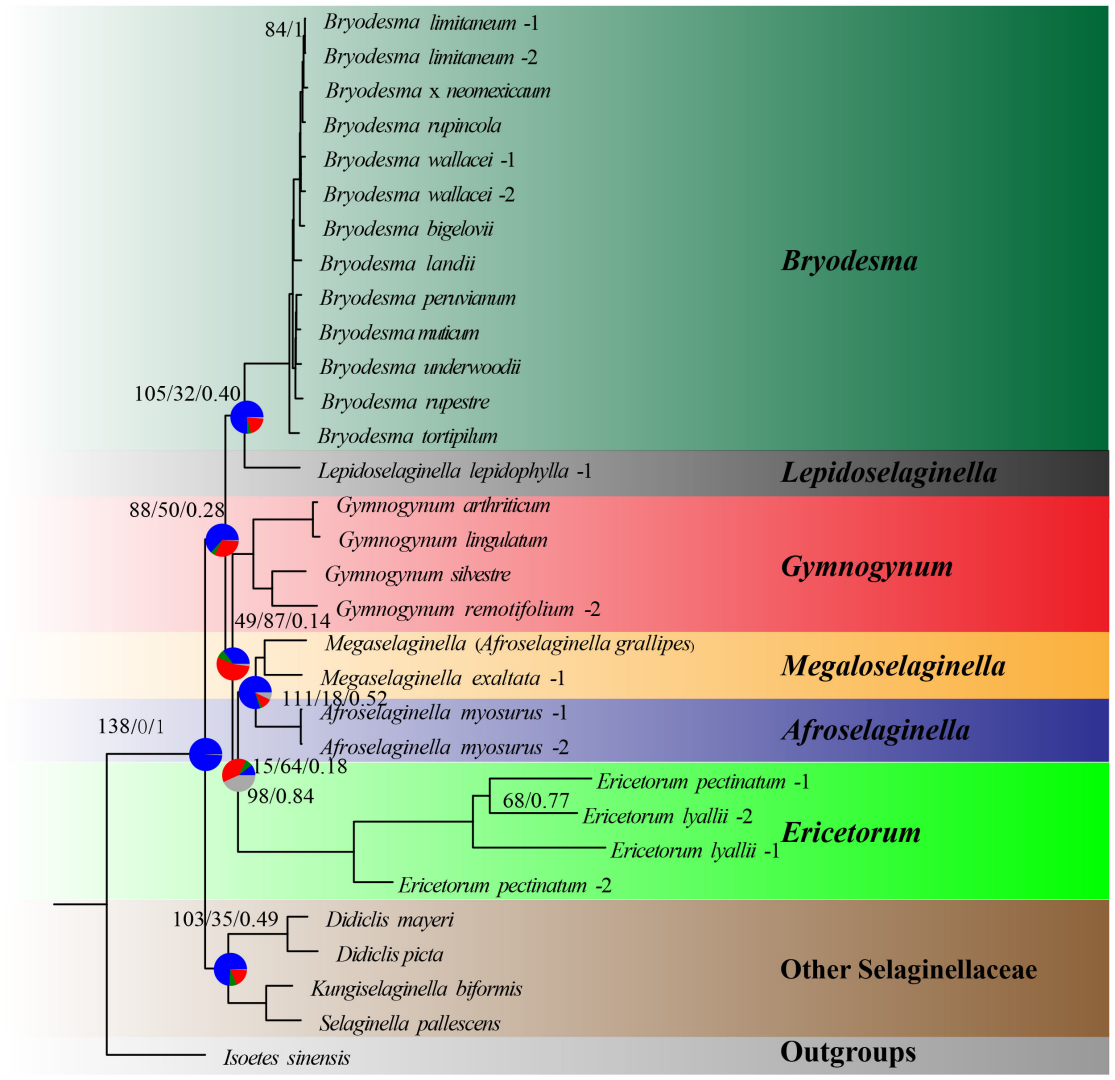
Maximum likelihood phylogeny of Selaginellaceae subf. Gymnogynoideae based on single-copy nuclear genes. Pie charts on the focused seven nodes, indicating the proportion of genes that agree (blue), support a main alternative topology (green), and support remaining alternatives (red) for a given node on the underlying topology. Numbers above the nodes indicate the number of concordant loci, the total number of conflicting loci, and the Internode Certainty All (ICA) scores. Maximum-likelihood bootstrap support values (MLBS) and Bayesian inference posterior probability (BIPP) are shown under the branches and are 100/1.0 unless otherwise indicated.

In total, 140 single-copy nuclear genes were used as baits to extract from our high-throught sequencing, but only 139 genes were used for further analyses because one gene failed to be assembled from reads (**Supplementary Figure S7**; **Supplementary Table S6**). In addition, fewer loci were successfully assembled for some old herbarium specimens with degraded DNA—for example, only 10 loci of the specimen *Ericetorum lyallii*-2, 24 loci of the specimen *E. lyallii*-1, 11 loci of the specimen *E. pectinatum*-1, and 63 loci of the specimen *E. pectinatum*-2 were assembled (**Supplementary Figure S7**; **Supplementary Table S6**). Both BI and ML methods were used to reconstruct tree species based on a concatenated single-copy genes (SCGs) supermatrix. In all analyses, all six genera of Gymnogynoideae were recovered as monophyletic and fully supported (MLBS = 100; BIPP = 1.0; **Figure 6**). The topology was the same as the ML topology based on all plastid datasets: *Bryodesma* was sister to *Lepidoselaginella* (MLBS = 100; BIPP = 1.0), and they together were sister to all other genera of Gymnogynoideae (**Figure 6**); *Gymnogynum* was sister to *Megaloselaginella* + *Afroselaginella* + *Ericetorum* with strong support (MLBS = 98; BIPP = 0.84), and *Ericetorum* was sister to *Megaloselaginella* + *Afroselaginella* with strong support (MLBS = 100; BIPP = 1.0; **Figure 6**). The combined data of the single-copy nuclear genes and each one of the plastid datasets resolved inter-generic relationships in Gymnogynoideae as follows: ((*Bryodesma*, *Lepidoselaginella*), (((*Megaloselaginella*, *Afroselaginella*), *Ericetorum*), *Gymnogynum*)), all relationships being fully supported except the sister relationship between *Ericetorum* and *Megaloselaginella* + *Afroselaginella* which received medium support (MLBS = 68–86; BIPP = 0.78–0.90; **Supplementary Figure S8**).

### 3.5 Discordance, ancestral state reconstruction, and network evolution

**Figure 7:**
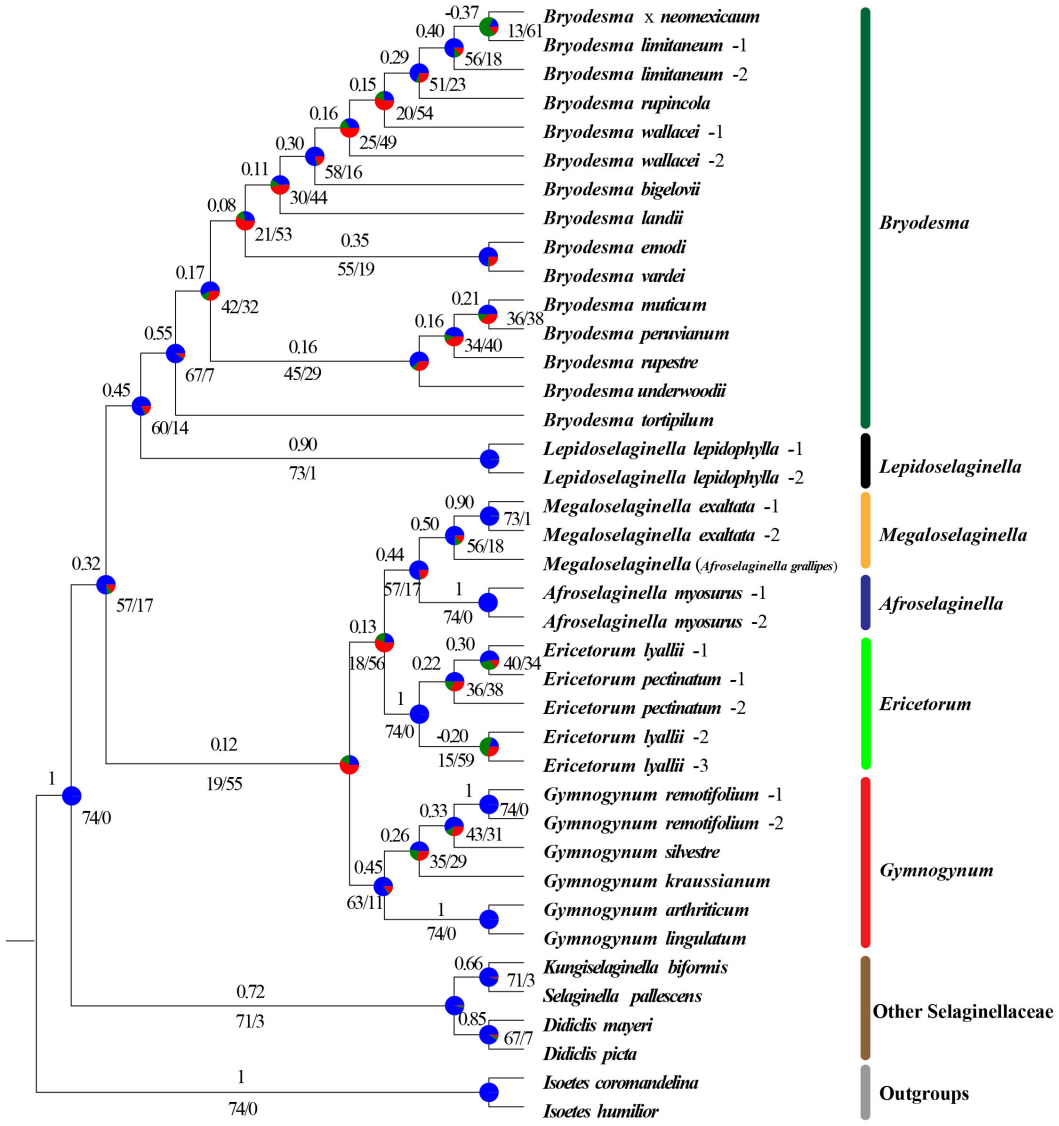
Concordant and conflict of the 74 loci on the maximum likelihood phylogeny. Pie charts indicate the proportion of genes that agree (blue), support a main alternative topology (green), and support remaining alternatives (red) for a given node on the underlying topology. Numbers above the nodes show the Internode Certainty All (ICA) scores, while the number under the nodes indicates the number of concordant loci and the total number of conflicting loci (support main alternative + support remaining alternatives).

The conflict analysis from Phyparts showed that less gene tree discordance was detected among nuclear genes and plastid genes regarding the placement of the six major clades (**Figures 6**, **7**). Phyparts suggested the monophyly of Gymnogynoideae with 138 out of 139 informative nuclear gene trees and all 74 plastid locus trees(**Figures 6**, **7**). The ICA values varied among the trees for the monophyly of the six genera each and for the relationships among them. The monophyly of *Bryodesma*, *Lepidoselaginella*, *Megaloselaginella*, *Afroselaginella*, *Ericetorum*, and *Gymnogynum* each was supported by 67 (ICA = 0.55), 73 (ICA = 0.90), 56 (ICA = 0.50), 74 (ICA = 1), 74 (ICA = 1), and 63 (ICA = 0.45) out of the 74 single-locus plastid trees (**Figure 7**), respectively. The clade comprising *Bryodesma* + *Lepidoselaginella* was supported by 105 nuclear gene trees (out of 139; ICA = 0.40; **Figure 6**) and 60 plastid loci trees (out of 74; ICA = 0.45; **Figure 7**), respectively. The clade comprising *Megaloselaginella* + *Afroselaginella* was supported by 111 nuclear gene trees (out of 139; ICA = 0.52; **Figure 6**) and 57 plastid loci trees (out of 74; ICA = 0.44; **Figure 7**), respectively. The sister relationship between the clade composed of *Bryodesma* + *Lepidoselaginella* and the clade composed of *Megaloselaginella* + *Afroselaginella* + *Ericetorum* + *Gymnogynum* was supported by 88 nuclear gene trees (out of 139; ICA = 0.28; **Figure 6**) and 57 plastid loci trees (out of 74; ICA = 0.32; **Figure 7**). However, the phylogenetic placement of *Ericetorum* and *Gymnogynum* showed a high proportion of discordance. The sister relationship between *Ericetorum* and the clade comprising *Megaloselaginella* + *Afroselaginella* was supported by only 15 nuclear gene trees (out of 139; ICA = 0.18; **Figure 6**) and only 18 plastid loci trees (out of 74; ICA = 0.13; **Figure 7**), while the sister relationship between *Gymnogynum* and the clade comprising *Ericetorum* + *Megaloselaginella* + *Afroselaginella* was supported by only 49 nuclear gene trees (out of 139; ICA = 0.14; **Figure 6**) and only 19 single-locus trees (out of 74; ICA = 0.12; **Figure 7**).

**Figure 8:**
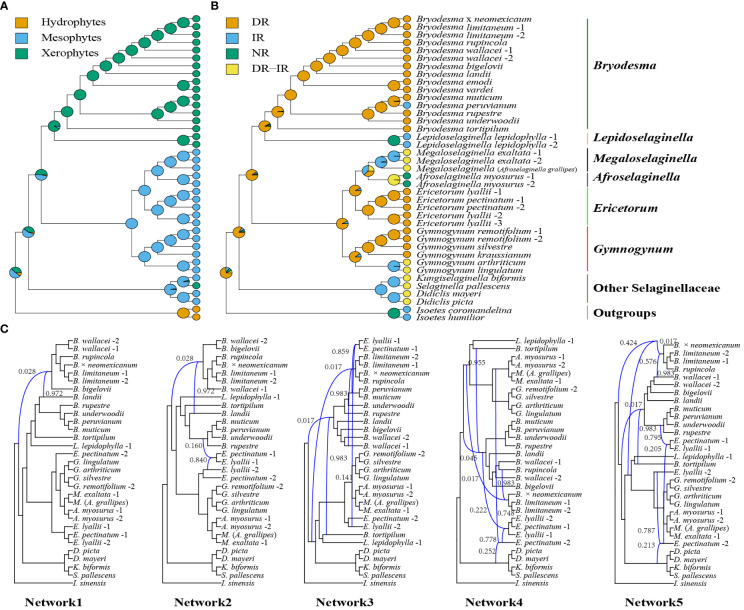
Ancestral state reconstruction of habit **(A)** and plastome master structures **(B)** in Selaginellaceae subf. Gymnogynoideae. The pie charts on nodes summarize the results of stochastic character mapping. The genus-level divisions are indicated behind the tip names. **(C)** Species network inferred from PhyloNet pseudolikelihood analyses with one to five hybridization events based on SCGs dataset. The curved branches indicate the minor and major edges of hybrid nodes, respectively. Numbers next to the curved branches indicate the inheritance probabilities for each hybrid node.

The character evolution of habits (hydrophytes, xerophytes, and mesophytes) and plastome master structures (DR, IR, NR, and DR–IR coexisting) is shown in **Figures 8A, B**. Both were not much homoplasious and thus useful to characterize the clades morphologically (**Figures 8A, B**). To analyze the potential causes of nuclear gene trees’ conflict, we employed SCGs datasets to perform a phylogenetic network analysis in PhyloNet while accounting for both hybridization and incomplete lineage sorting (ILS). One reticulation event from ancestral Selaginellaceae and *Bryodesma* lineage was inferred in all five examinations (**Figure 8C**), with an inheritance probability of 0.017 from the ancestral Selaginellaceae lineage and 0.983 from the *Bryodesma* lineage (**Figure 8C**). Four inferred hybridization events between *Bryodesma* and *Ericetorum* were also detected (**Figure 8C**: network 2−network 5).

## 4 Discussion

### 4.1 The relationship between *Afroselaginella* and *Megaloselaginella*

Although the ML trees are somewhat discordant with the BI trees based on plastid datasets, our phylogenetic inference strongly supported the sister relationship between *Afroselaginella* and *Megaloselaginella* in all analyses based on both plastid and nuclear loci (**Figures 2**–**7**; **Supplementary Figures S1**-**S5**). Based on two molecular makers (*rbcL* + ITS; Zhou et al., 2016) and morphological difference, *Megaloselaginella* (including *M. exaltata*) and *Afroselaginella* (including *A. myosurus*) were recognized by Zhou and Zhang (2015), albeit as sections of their *S.* subg. *Gymnogynum*. Subsequently, Weststrand and Korall (2016b) combined the two taxa and coined *S.* subg. *Exaltatae* based on the Bayesian analysis of three genes (*rbcL* + *pgiC* + *SQD1*; Weststrand and Korall, 2016a, b). Several previous phylogenomic studies of Selaginellaceae involved Gymnogynoideae (e.g., Du et al., 2020; Zhang et al., 2020a), but *Afroselaginella* was still not sampled. Here we provide the first strong phylogenomic evidence that *Afroselaginella* is indeed sister to *Megaloselaginella* with full support (**Figures 2**–**7**; **Supplementary Figures S1**-**S4**, **S8**).

Despite the confirmed sister relationship between *Afroselaginella* and *Megaloselaginella*, considering the distinction between them, here we support the recognition of both at generic rank. Firstly, species of *Megaloselaginella* have large and erect plants, articulate stems, and pillared to baculite microspore surfaces (Zhou and Zhang, 2015; Weststrand and Korall, 2016a, b; Zhou and Zhang, 2023), whereas those of *Afroselaginella* have medium-sized and creeping plants, inarticulate stems and branches, and microspores with an equatorial ring and verrucate surfaces (Zhou and Zhang, 2015; Weststrand and Korall, 2016a, b; Zhou and Zhang, 2023). In previous studies, *Megaloselaginella* was generally thought to be only distributed in the Central and South America, whereas those of *Afroselaginella* only in western Africa (Zhou and Zhang, 2015, 2023). However, this phylogenetic analysis showed that the western Africa species *Afroselaginella grallipes* was clustered into *Megaloselaginella*. We further studied the specimen of *Afroselaginella grallipes* and confirmed that it is indeed a member of *Megaloselaginella*. This species with large plants and articulate stems is highly consistent with those members of *Megaloselaginella*. *Afroselaginella grallipes* should be transferred to *Megaloselaginella*. *Afroselaginella* has the smallest plastome size (ca. 100 kb) in Gymnogynoideae (**Supplementary Table S5**) and a unique plastome structure with only SC region but without a repeat region and lacking one ribosomal operon in Selaginellaceae (**Supplementary Table S5**), whereas *Megaloselaginella* has DR–IR coexisting plastome structure with three conformations *in vivo*. In addition, molecular dating showed that *Afroselaginella* and *Megaloselaginella* diverged from each other ca. 210 Ma (Klaus et al., 2017), an age older than the stem ages of most of the fern families recognized by PPG I (2016) following the molecular datings by Schuettpelz and Pryer (2009); Rothfels et al. (2015), and Testo and Sundue (2016). Similar arguments also apply to the recognition of other four genera in Gymnogynoideae.

### 4.2 Phylogenetic signals of plastid loci for incongruent nodes

Regarding *Ericetorum* and *Gymnogynum*, our concordance analysis detected an obvious discordance among trees based on different plastid loci, and ML and BI trees based on all plastid loci datasets demonstrated a high proportion of conflict (**Figures 2**–**5**, **7**; **Supplementary Figures S1**-**S4**). The topologies of BI trees are identical to one another based on all datasets after removing the outlier loci (**Supplementary Figures S2**-**S4**). The topologies of BI trees generally did not change obviously based on any datasets, although BIPP values varied, even after the outlier loci were removed (**Supplementary Figures S2**-**S5**). To assess the alternative placements of *Ericetorum* and *Gymnogynum*, we used the approaches of Shen et al. (2017) to measure the phylogenetic signal of each plastid locus. As previous studies had shown, conflicts among different sequence types were also detected [coding (gene) vs. non-coding regions (intergene)] (Zhang et al., 2020b; Yang et al., 2021; Zhang et al., 2022). For the alternative resolutions of *Ericetorum*, except T2 with a higher proportion of ΔGLS (35%) in the intergene dataset, the remaining two fundamental datasets (codon12 and gene datasets) showed that T1 had higher ΔGLS (41%) (**Figure 4D**). Removing the outlier loci, all the derived four datasets (codon12_RE, gene_RE, codon12_REG, and gene_REG) showed that T1 had a relatively higher proportion of ΔGLS (41%–44%), but T1 and T2 had the same high proportion in intergene_RE dataset (38%) and intergene_REG dataset (39%), respectively (**Figure 4D**). For *Gymnogynum*, T4 had a higher proportion of ΔGLS (39%) in the intergene dataset, T1 had a higher proportion of ΔGLS (33%–37%) in codon12, codon12_RG, and codon12_REG datasets, and T2 had a relatively higher proportion of ΔGLS (31%) in gene, gene_RG, and gene_REG datasets (**Figure 5D**). For the T1 of *Gymnogynum*, maybe the actual positions had higher summarized ΔGLS and ΔSLS proportions (**Figures 5B, C**). The different support values (T1 vs. T2) of *Gymnogynum* (codon12/codon12_RG/codon12_REG vs. gene/gene_RG/gene_REG) also demonstrated that the selection of codon positions might affect the accuracy and precision of phylogeny (Simmons et al., 2006b). Moreover, ΔGLS had a decrease in different degrees based on gene_intergene datasets-concatenated coding and non-coding regions, but the T1 of *Ericetorum* and *Gymnogynum* had a higher proportion of ΔGLS and ΔSLS with gene datasets (**Figures 4**, **5**). It could be caused by the difference in evolutionary force in the coding and non-coding regions (Gielly and Taberlet, 1994).

The ΔGLS is correlated with the gene length of plastid genes, and the topology supported by the largest number of sites (ΔSLS) was further used to assess the variation of the phylogenetic signal. All datasets showed that T1 of *Ericetorum* had a higher proportion of ΔSLS (**Figure 4E**), but T2 of *Ericetorum* had a higher proportion of ΔGLS based on intergene datasets (**Figure 4D**). Because noises are randomly distributed in sequences (Delsuc et al., 2005), those data with strong signals rather than simply with more loci should be selected and used in phylogenetic inference. Recent studies have discovered that plastid genes were largely uninformative in rosids, Fagales, and Leguminosae (Gonçalves et al., 2019; Koenen et al., 2020; Zhang et al., 2020b; Yang et al., 2021). However, our concordance analyses showed that, except for each node of *Ericetorum* and *Gymnogynum*, almost all plastid loci provided a high proportion of trees that are congruent with one another for the remaining nodes (**Figure 7**). Nevertheless, after removing the outliers, the phylogenetic signals of most possible topologies in each derived dataset generally increased in various proportions (**Figures 3**–**5**). Our discordance and phylogenetic signal analysis show that both T1 of *Ericetorum* and that of *Gymnogynum* are most strongly supported by plastid data. Although the sources of conflict in plastome phylogenies are poorly understood, in this study they can be a combination of the unique plastome traits, high substitution rate, high level of rate heterogeneity, missing data, high GC content, unusual RNA editing, long evolutionary history, the analysis methods (ML, BI) themselves, etc. The extremely high substitution rates and high level of rate heterogeneity have been reported for the plastid genes of Selaginellaceae, which could cause issues in phylogenetic inference (Korall and Kenrick, 2004; Zhang et al., 2020a). Plastomes of Selaginellaceae generally have a high GC content (>50%) (**Supplementary Table S5**) (Tsuji et al., 2007; Smith, 2009; Xu et al., 2018; Du et al., 2020; Zhang et al., 2020a, 2021; Zhang and Zhang, 2022, 2022; Xiang et al., 2022; Yang et al., 2022; Zhou et al., 2022; Tang et al., 2023), which was thought to be correlated with a high level of RNA editing (Du et al., 2020; Xiang et al., 2022; Yang et al., 2022) The plastome of *Didiclis uncinata* (Desv. ex Poir.) Li Bing Zhang & X.M. Zhou, a member of the same family, was reported to have 3,415 RNA editing sites (Oldenkott et al., 2014). Extreme plastid RNA editing may confound the phylogenetic reconstruction (Du et al., 2020; Zhang et al., 2022). Because old herbarium materials were used in sequencing, a few critical plastomes have quite a bit of missing data—for example, the four samples of *Ericetorum* have 10–63 missing loci (**Supplementary Figure S7**; **Supplementary Table S6**). This added the uncertainty to the phylogenetic estimates (Philippe et al., 2000; Goremykin et al., 2010). A single search and holding only a single tree in ML analysis have been known for spurious inferences, and BI has been known for inflated support values under various circumstances (Simmons et al., 2004, 2006a, Simmons and Freudenstein, 2011). In addition, our analyses showed that there was a very high level of heterogeneity among single-copy nuclear gene trees (**Figure 6**). While these inconsistencies have never been well explained, it is often believed that the phylogenetic incongruence in plants is mainly due to hybridization (Liu et al., 2022; Zhao et al., 2023). We also detected inter-genus geneflow among almost all lineages of Gymnogynoideae (**Figure 8C**), so that topology conflict in Gymnogynoideae at the genus level was potentially caused by hybridization. All of these factors might have acted simultaneously and/or cumulatively contributing to the observed conflicts in phylogenetic inference in this study.

### 4.3 The overall phylogeny in Gymnogynoideae

In this study, the monophyly of six genera in Gymnogynoideae is confirmed with strong support based on plastid data (MLBS = 100; BIPP = 1.0; ICA = 0.44–1.0; **Figures 2**, **7**; **Supplementary Figures S1**-**S4**). In previous studies based on complete or nearly complete plastomes (Du et al., 2020; Zhang et al., 2020a; Zhou et al., 2022), the relationships among the genera of Gymnogynoideae were debatable depending on datasets (**Figure 1**). Part of the reasons may be attributed to the limited taxon samplings (fewer than eight accessions), particularly, *Afroselaginella* was never sampled before in any phylogenomic studies (Du et al., 2020; Zhang et al., 2020a; Zhou et al., 2022).

Using expanded taxon and character sampling and comprehensive analytical methods for dissecting signal and conflict among loci, despite some existing conflicting plastid trees, the most plausible overall phylogeny in Gymnogynoideae appears to be ((*Bryodesma*, *Lepidoselaginella*), (((*Megaloselaginella*, *Afroselaginella*), *Ericetorum*), *Gymnogynum*)) (**Figure 2A**). This topology is not only supported by most plastid evidence/tree topologies but also is consistent with the nuclear tree based on single-copy nuclear genes (**Figure 6**). *Bryodesma* is resolved as sister to *Lepidoselaginella* (**Figures 1**, **2**, **7**; **Supplementary Figures S1**-**S4**), consistent with the results of most previous studies (Arrigo et al., 2013; Zhou et al., 2016; Weststrand and Korall, 2016a; Klaus et al., 2017; Du et al., 2020; Zhang et al., 2020a). *Bryodesma* + *Lepidoselaginella* together are sister to the rest of the subfamily, in accordance with the results of Weststrand and Korall (2016a) based on *rbcL*, *pgiC*, and *SQD1* data and those of Zhou et al. (2016) based on *rbcL* and ITS data, although both latter two studies provided weak support for this resolution. *Gymnogynum* is sister to the remaining three genera together, and *Ericetorum* is sister to *Megaloselaginella* + *Afroselaginella*, in agreement with the results of Weststrand and Korall (2016a) and Zhou et al. (2016), too; again, the latter two provided weak support. Overall, our large plastid and nuclear datasets independently well resolved the relationships in the subfamily, consistent with the two most recent large phylogenies (Zhou et al., 2016; Weststrand and Korall, 2016a) based on Sanger sequence data, and we here provide the strongest support for these relationships for the first time. Notably, based on six plastid and nuclear markers, Zhou and Zhang (2023) resolved *Ericetorum* as sister to *Gymnogynum*, but the support values were low (<50%).

### 4.4 Evolution of habit and plastome master structures in Gymnogynoideae

With the relationships well resolved in the subfamily, we can understand how habit and plastome master structures evolved in Gymnogynoideae. The ancestral state of the habit of clade A (*Bryodesma* + *Lepidoselaginella*) was xerophytes (or “resurrection”) and that of clade B (*Megaloselaginella*, *Afroselaginella*, *Ericetorum*, and *Gymnogynum*) was mesophytes (**Figure 8A**), but the ancestral state of the habit of Gymnogynoideae had nearly the same proportion of xerophytes and mesophytes (**Figure 8A**). Reconstruction of plastome master structures showed that DR was the ancestral state of Gymnogynoideae, and the other three (IR, NR, and DR–IR) were merged with the evolution of plastomes (**Figure 8B**).

## Data availability statement

The new sequenced chloroplast genome of this study has been submitted to GenBank under the accession number of PP693476 to PP693484, respectively. Multiple sequence alignments, phylogenetic trees, trait ancestral character state reconstructions, are available on the Figshare repository: 10.6084/m9.figshare.25750449.v1.

## Author contributions

JZ: Writing – original draft, Software, Methodology, Formal analysis, Conceptualization. ZRH: Writing – review & editing, Conceptualization. SLF: Writing – original draft, Software, Methodology. XKH: Writing – original draft. LYJ: Writing – original draft. YPH: Writing – original draft. HY: Writing – review & editing, Conceptualization. LBZ: Writing – review & editing, Conceptualization. XMZ: Writing – review & editing, Conceptualization, Founding acquisition.

## Supplementary material

Supplementary Figure 1 Maximum likelihood phylogeny of Selaginellaceae subf. Gymnogynoideae based on different datasets. Maximum likelihood bootstrap support values (MLBS) are shown above the branches and are 100/1.0 unless otherwise indicated. The MLBS from left to right were based on the dataset of codon12, gene, intergene, gene_intergene, codon12_RE, gene_RE, intergene_RE, gene_intergene_RE, codon12_RG, gene_RG, intergene_RG, gene_intergene_RG, codon12_REG, gene_REG, intergene_REG, and gene_intergene_REG, respectively.

Supplementary Figure 2 Phylogenetic tree reconstruction using Bayesian inference (BI) based on the dataset of codon12, codon12_RE, codon12_RG, and codon12_REG, respectively. Numbers above the branches represent Bayesian inference posterior probability (BIPP) and are 1.0/* unless otherwise indicated at the nodes.

Supplementary Figure 3 Phylogenetic tree reconstruction using Bayesian inference (BI) based on the dataset of intergene, intergene_RE, intergene_RG, and intergene_REG, respectively. Numbers above the branches represent Bayesian inference posterior probability (BIPP) and are 1.0/* unless otherwise indicated at the nodes.

Supplementary Figure 4 Phylogenetic tree reconstruction using Bayesian inference (BI) based on the dataset of gene, gene_intergene, gene_RE, gene_intergene_RE, gene_RG, gene_intergene_RG, gene_REG, and gene_intergene_REG, respectively. Numbers above the branches represent Bayesian inference posterior probability (BIPP) and are 1.0/* unless otherwise indicated at the nodes.

Supplementary Figure 5 Three alternative topologies for the *Ericetorum* (red) placement in the Selaginellaceae subf. Gymnogynoideae.

Supplementary Figure 6 Four alternative topologies for the *Gymnogynum* (red) placement in the Selaginellaceae subf. Gymnogynoideae.

Supplementary Figure 7 HybPiper recovery heatmap to visualize the recovery efficiency. Each row shows a sample, and each column is a gene. The amount of shading in each box corresponds to the length of the gene recovered for that sample, relative to the length of the reference.

Supplementary Figure 8 Maximum likelihood phylogeny of Selaginellaceae subf. Gymnogynoideae based on the datasets of SCGs_codon12, SCGs_gene, SCGs_intergene, and SCGs_gene_intergene. Maximum-likelihood bootstrap support values (MLBS) and Bayesian inference posterior probability (BIPP) are shown above the branches and are 100/1.0 unless otherwise indicated.

Supplementary Table 1 List of plastomes used in this study. Subgenus *Gymnogynum* in the row of “Subgenus (Zhou and Zhang, 2015)” is identical to *S.* subg. *Ericetorium* Jerm *sensu* Zhou and Zhang (2015).

Supplementary Table 2 Best-fitting likelihood model for maximum likelihood (ML) and Bayesian inference analyses (BI) for each dataset in this study.

Supplementary Table 3 List of the loci in each dataset.

Supplementary Table 4 Character coding of the taxa used in this study.

Supplementary Table 5 Features of plastomes used in this study.

Supplementary Table 6 Length of sequence recovered by HybPiper for each single-copy gene and sample in this study.
