# Supplementary figures and images for: Phylogenomic data resolved the deep relationships of Gymnogynoideae (Selaginellaceae)
Source: Front Plant Sci. 2024 Jul 16;15:1405253. doi: 10.3389/fpls.2024.1405253 (PMC11287774; doi:10.3389/fpls.2024.1405253)

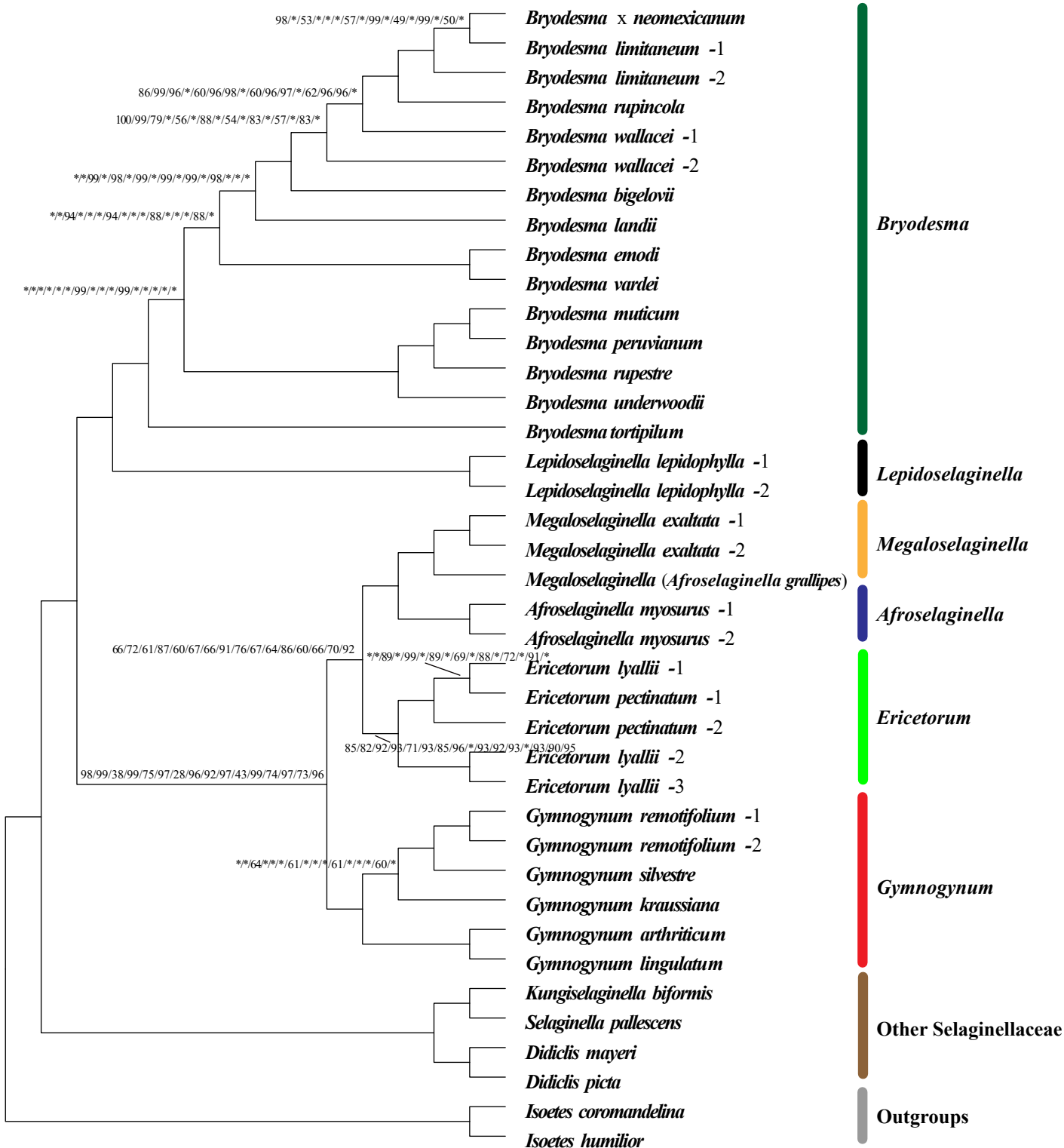

Supplement: Supplementary Figure 1 — Maximum likelihood phylogeny of Selaginellaceae subf. Gymnogynoideae based on different datasets. Maximum likelihood bootstrap support values (MLBS) are shown above the branches and are 100/1.0 unless otherwise indicated. The MLBS from left to right were based on the dataset of codon12, gene, intergene, gene_intergene, codon12_RE, gene_RE, intergene_RE, gene_intergene_RE, codon12_RG, gene_RG, intergene_RG, gene_intergene_RG, codon12_REG, gene_REG, intergene_REG, and gene_intergene_REG, respectively. [file Image_1.pdf]

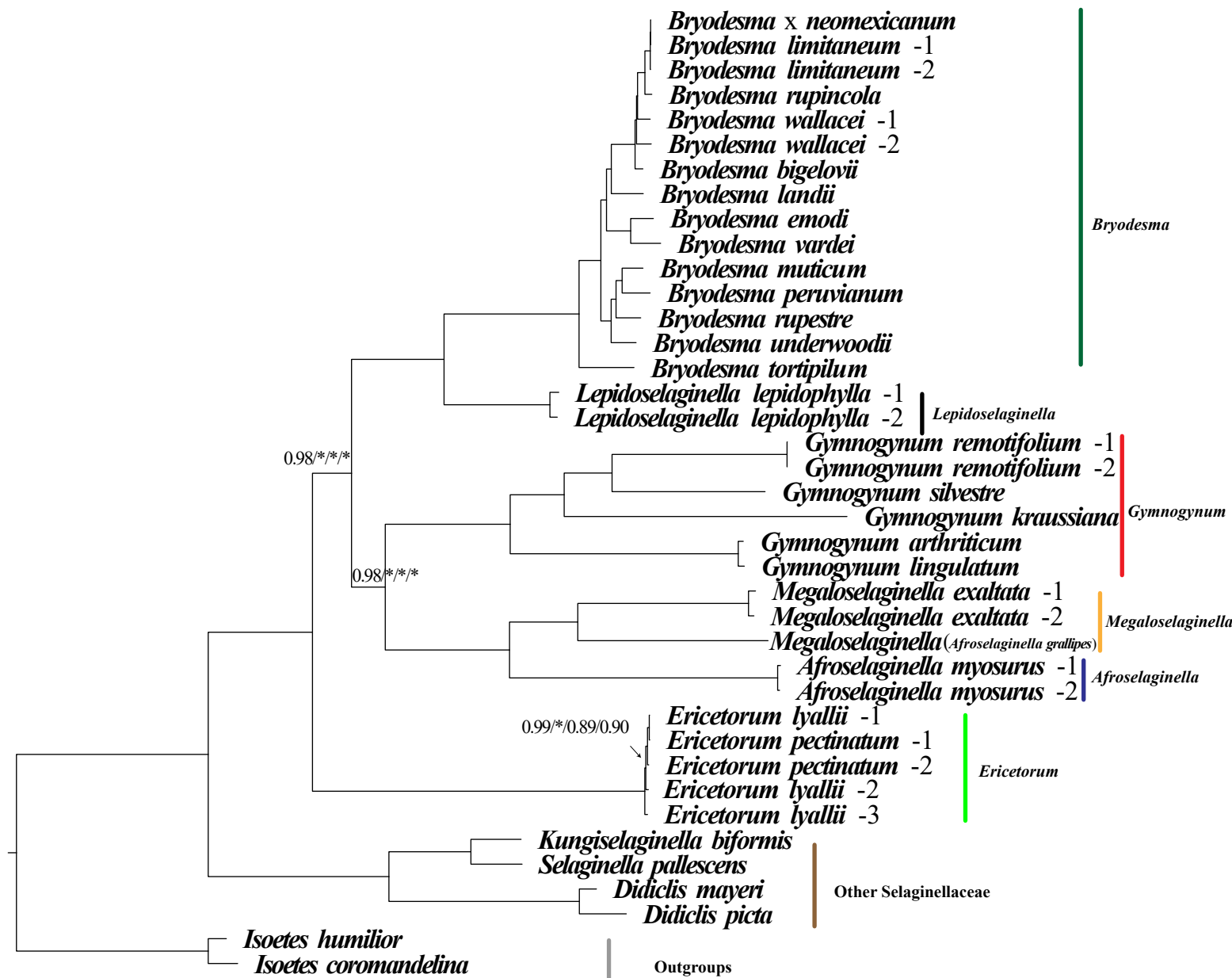

Supplement: Supplementary Figure 2 — Phylogenetic tree reconstruction using Bayesian inference (BI) based on the dataset of codon12, codon12_RE, codon12_RG, and codon12_REG, respectively. Numbers above the branches represent Bayesian inference posterior probability (BIPP) and are 1.0/* unless otherwise indicated at the nodes. [file Image_2.pdf]

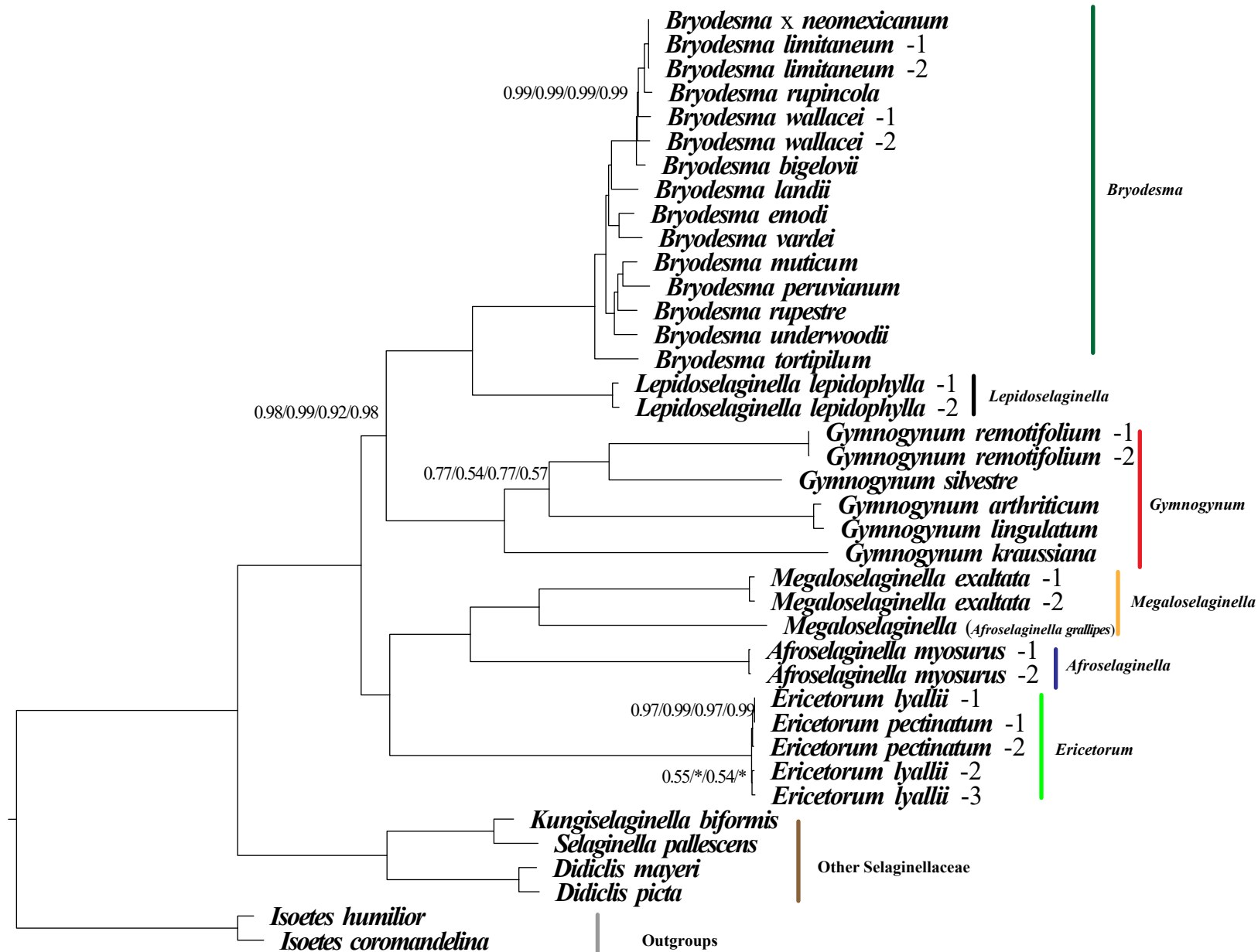

Supplement: Supplementary Figure 3 — Phylogenetic tree reconstruction using Bayesian inference (BI) based on the dataset of intergene, intergene_RE, intergene_RG, and intergene_REG, respectively. Numbers above the branches represent Bayesian inference posterior probability (BIPP) and are 1.0/* unless otherwise indicated at the nodes. [file Image_3.pdf]

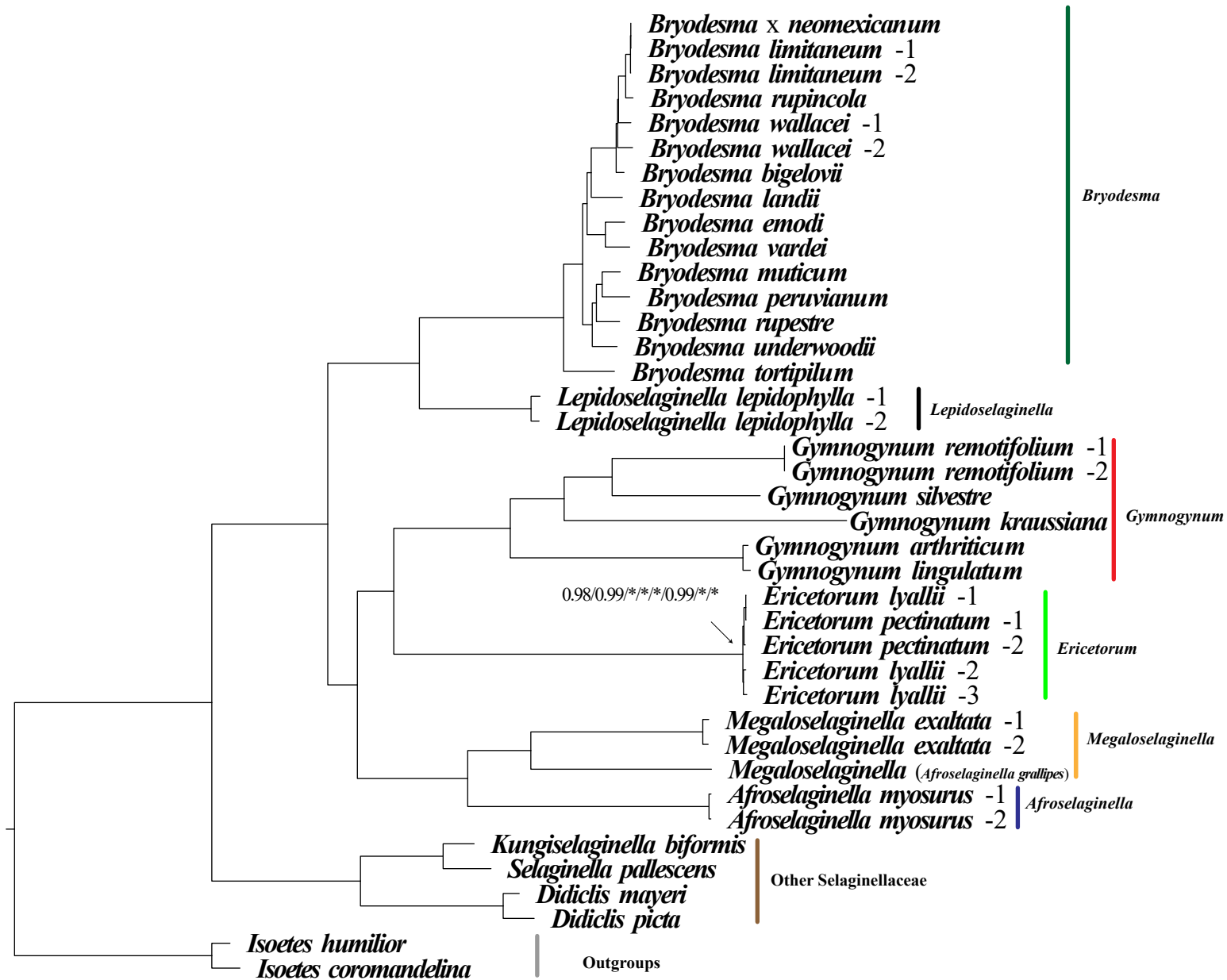

Supplement: Supplementary Figure 4 — Phylogenetic tree reconstruction using Bayesian inference (BI) based on the dataset of gene, gene_intergene, gene_RE, gene_intergene_RE, gene_RG, gene_intergene_RG, gene_REG, and gene_intergene_REG, respectively. Numbers above the branches represent Bayesian inference posterior probability (BIPP) and are 1.0/* unless otherwise indicated at the nodes. [file Image_4.pdf]

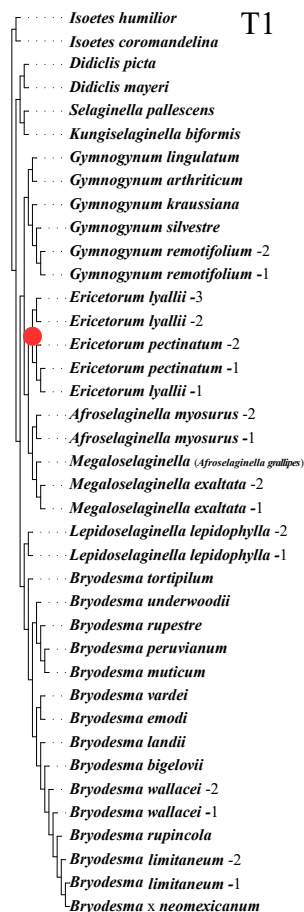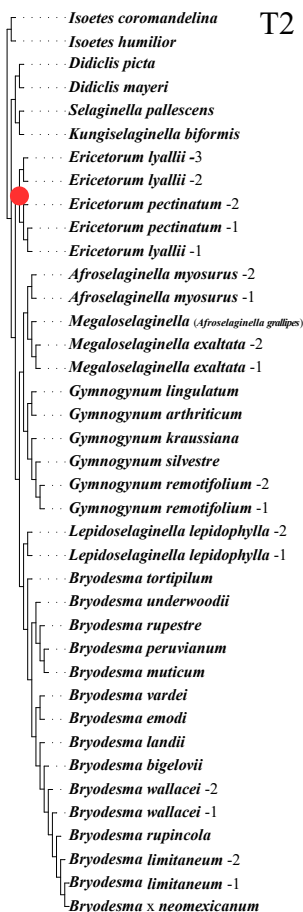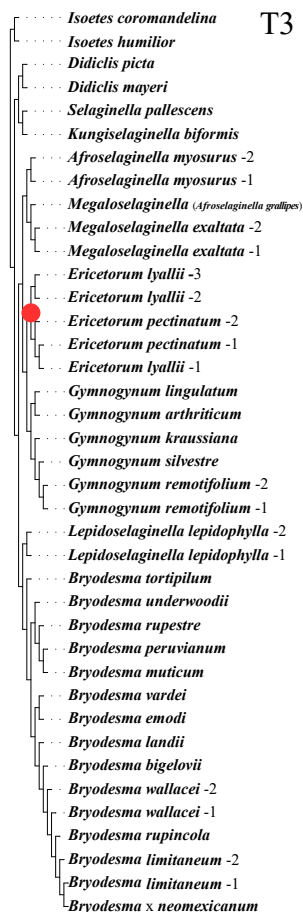

Supplement: Supplementary Figure 5 — Three alternative topologies for the Ericetorum (red) placement in the Selaginellaceae subf. Gymnogynoideae. [file Image_5.pdf]

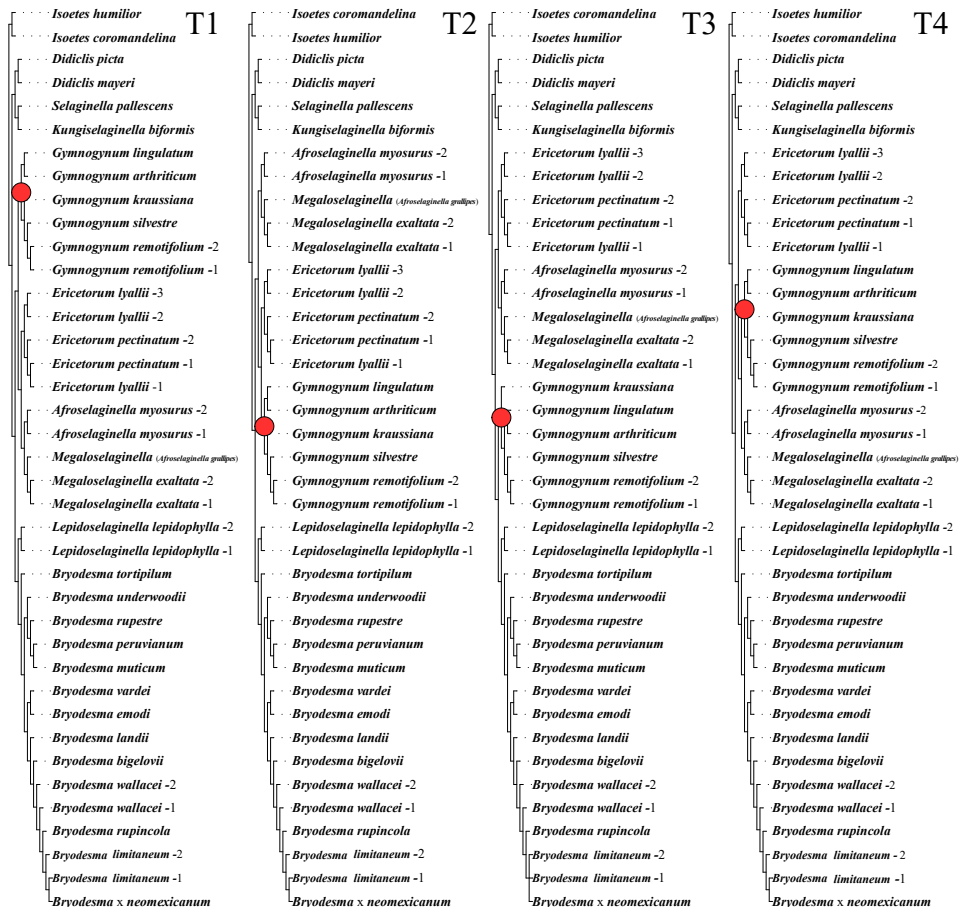

Supplement: Supplementary Figure 6 — Four alternative topologies for the Gymnogynum (red) placement in the Selaginellaceae subf. Gymnogynoideae. [file Image_6.pdf]

Percentage length recovery for each gene, relative to mean of targetfile references

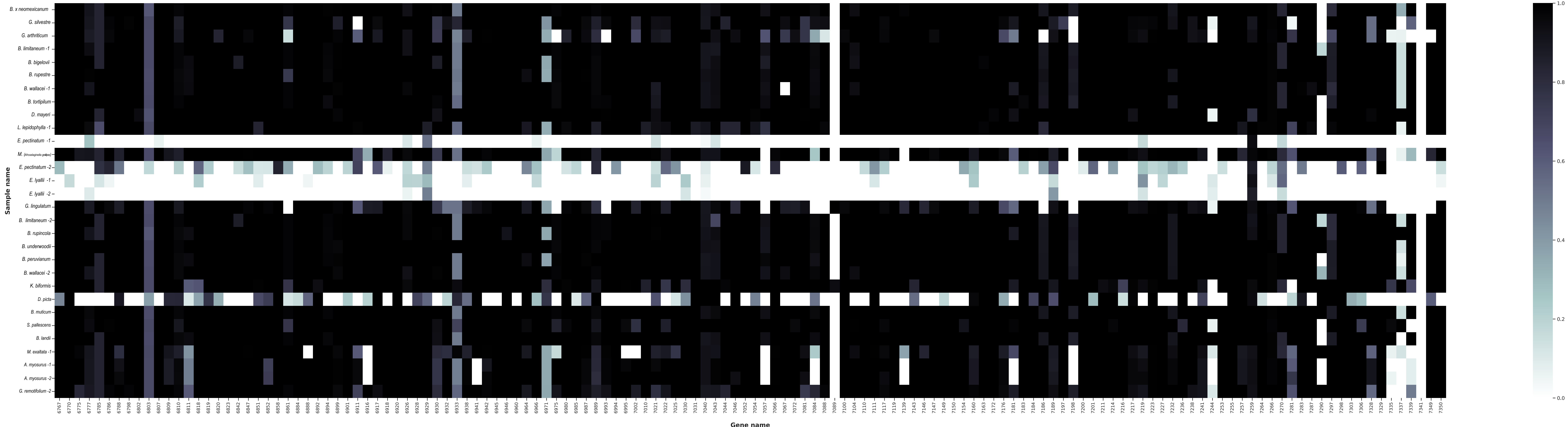

Supplement: Supplementary Figure 7 — HybPiper recovery heatmap to visualize the recovery efficiency. Each row shows a sample, and each column is a gene. The amount of shading in each box corresponds to the length of the gene recovered for that sample, relative to the length of the reference. [file Image_7.pdf]

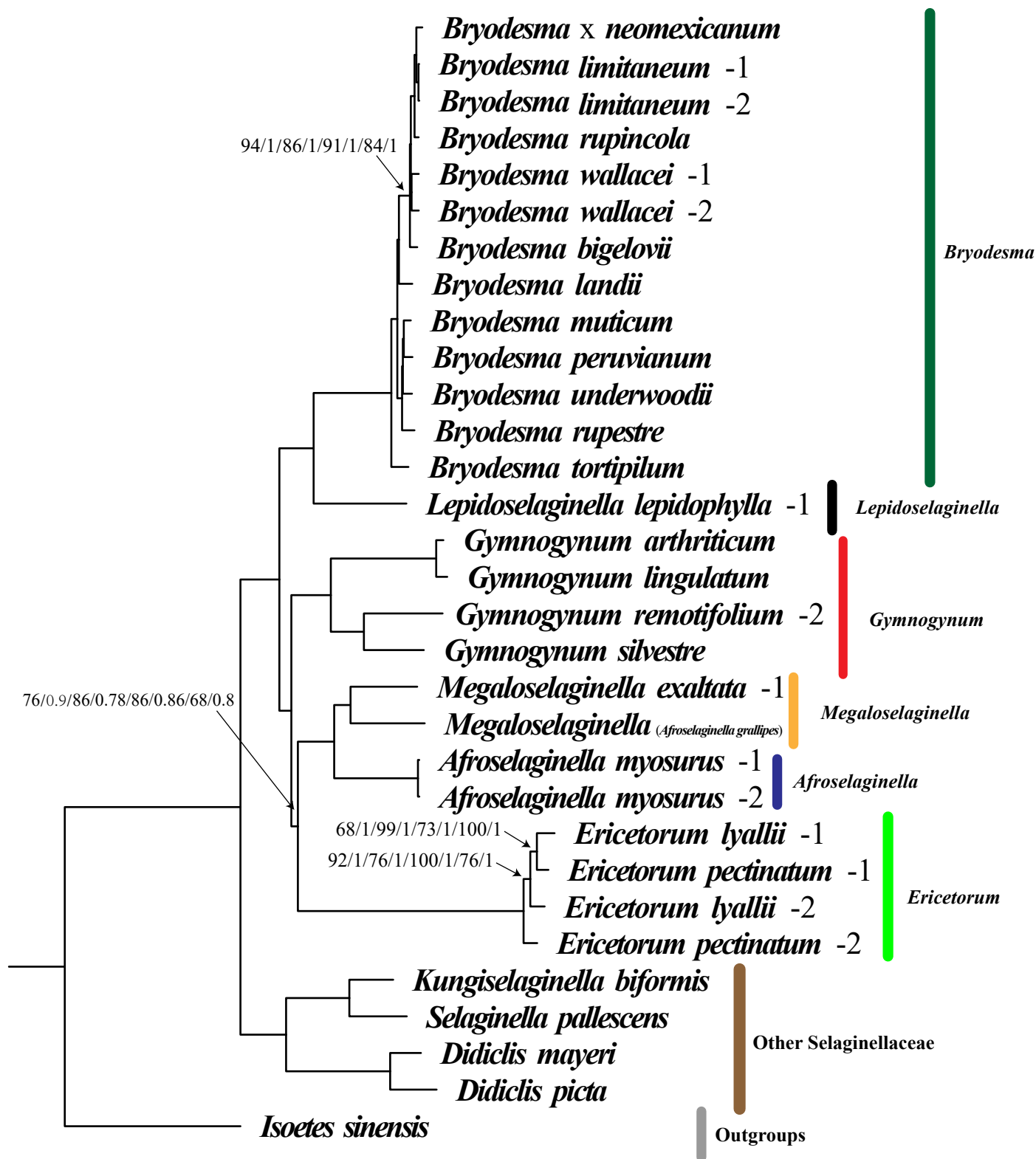

Supplement: Supplementary Figure 8 — Maximum likelihood phylogeny of Selaginellaceae subf. Gymnogynoideae based on the datasets of SCGs_codon12, SCGs_gene, SCGs_intergene, and SCGs_gene_intergene. Maximum-likelihood bootstrap support values (MLBS) and Bayesian inference posterior probability (BIPP) are shown above the branches and are 100/1.0 unless otherwise indicated. [file Image_8.pdf]
